# Cross Talk between Inflammation and Metabolic Disorders

**DOI:** 10.1155/2022/9821506

**Published:** 2022-04-13

**Authors:** Jie Chen, Yan Yang, Wen Kong

**Affiliations:** ^1^Jiangxi Provincial People's Hospital Affiliated to Nanchang University, Nanchang, China; ^2^Jiangxi Provincial People's Hospital, Nanchang Medical College, Nanchang, China; ^3^Rutgers New Jersey Medical School, Newark, USA; ^4^Wuhan Union Hospital, Tongji Medical College, Huazhong University of Science and Technology, Wuhan, China

Metabolic disorders are a number of diseases including atherosclerosis, diabetes, obesity, gout, rheumatoid arthritis (RA), osteoporosis, and osteopenia. Acute or chronic inflammatory processes often coexist with occurrence and development of these diseases, and to date, many studies have implied that metabolic diseases are associated with inflammation. Furthermore, in some studies, the related inflammatory cells and molecules are also confirmed. For example, type 2 diabetes has been wildly considered an inflammatory disease with inflammatory or anti-inflammatory cells including CD11b^+^CD11c^+^ myeloid cells and CD3^+^T cells and cytokines such as IL-1*β* [1, 2]. RA has long been believed as a chronic inflammatory joint disease characterized by persistent synovitis, systemic inflammation, and autoantibodies, and reducing synovitis and systemic inflammation by disease-modifying antirheumatic drugs can effectively improve joint function [3]. Acute inflammation of gout is mediated principally by macrophages, neutrophils, and inflammatory cytokines including IL-1*β* in response to monosodium urate crystals [4]. Although the association between the inflammation and metabolic disorders has been continuously reported, the inflammatory mechanism and involved factors of the pathogenesis of these diseases still need to be further clarified. This special issue consists of a number of original research articles and review papers on the topic of cross talk between inflammation and metabolic disorders.

Cytokines, both inflammatory and anti-inflammatory, play vital roles in the process of acute or chronic inflammation [5, 6]. A research by Himmerich et al. focused on patients with anorexia nervosa and found that several cytokines including GM-CSF, MCP-4, and IL-4 were positively associated with all three parameters of body water (intracellular, extracellular, and total body water), whereas IFN-*γ*, IL-6, and IL-10 were negatively associated with all the parameters. This finding suggests an interaction between body water and the cytokine system, confirming the existence of the underlying mechanism related to anorexia nervosa-associated inflammatory processes.

Risk factors, including dietary, environmental, and habitual factors, are considered to be involved in the cause and progress of some metabolic diseases [7, 8]. Moreover, for some certain metabolic disorders, other coexisting metabolic diseases are also risk factors. For instance, except for dietary factors, obesity is also a risk factor for gout [9], indicating a deep relationship on the mechanism between the two or more diseases. Li et al. analyzed the factors that are related to the serum uric acid levels and found significant differences between gout with coronary heart disease patients and gout with cerebral infarction patients. They also discovered a history of smoking as a risk factor that affects the therapeutic effects of young gout with cerebral infarction patients. Wang et al. determined that a higher phosphorus value and higher alkaline phosphatase concentration and a longer use of glucocorticoids were risk factors for bone mass loss in kidney transplant recipients and implied that maintaining an appropriate weight and exercising appropriately may help to maintain bone mass.

During the process of inflammation, immune cells play vital roles in promoting or regulating the progress of inflammatory response. Macrophages, comprising of proinflammatory M1 and anti-inflammatory M2 group, are reported to be associated with many inflammatory diseases such as obesity, asthma, and atherosclerosis [10]. In this special issue, Yang et al. reported that by regulating the polarization of macrophages toward M2, GLP-1R has a protective effect in the progression of coronary atherosclerosis. Wang et al. were also focused on M2 polarization and found a protective mechanism related to miR-6869-5p and protein tyrosine phosphatase receptor type O in gestational diabetes mellitus. Moreover, in a study by Su et al. focused on therapy of obese complicated with polymicrobial sepsis by exploring a mouse model, macrophages were found to be involved in the mechanism by which administrated glutamine alleviated inflammation and attenuated acute kidney injury. Except for cells, a big group of inflammatory molecules also exist and are involved in inflammation. Peng et al. summarized how CCN3, a member of the CCN proteins which is a family of extracellular matrix-associated proteins, acts in immune-related metabolic diseases and the related mechanisms.

Antivirus immune response upon infection results with an inflammatory process. Chronic cytomegalovirus (CMV) infection significantly irritates the immune system and has been found to be one of the main determinants of immune senescence in the elderly [11, 12]. Accordingly, Lin et al. investigated the relationship between CMV infection and leukocyte telomere length and found a close relevance between previous CMV infection and shorter leukocyte TL. Novel coronavirus disease 2019 (COVID-19) caused by severe acute respiratory syndrome coronavirus 2 is a deadly disease due to the status of hyperinflammation after infection [13]. Zheng et al. investigated the potential predictors of COVID-19 mortality and risk factors for hyperinflammation in COVID-19 and found uric acid levels to be a potential factor associated with inflammatory markers.

Collectively, all the original research and review articles in this special issue cover many important aspects in the area of interaction between inflammation and metabolic disorders, which may provide new strategies for the diagnosis and treatment of metabolic diseases in the clinic.
